# Response to Radioiodine Therapy for Thyrotoxicosis: Disparate Outcomes for an Indigenous Population

**DOI:** 10.1155/2016/7863867

**Published:** 2016-06-29

**Authors:** Jade A. U. Tamatea, John V. Conaglen, Marianne S. Elston

**Affiliations:** ^1^University of Auckland, Waikato Clinical Campus, Private Bag 3200, Hamilton 3240, New Zealand; ^2^Department of Endocrinology, Waikato Hospital, Private Bag 3200, Hamilton 3240, New Zealand

## Abstract

Despite 70 years of experience treating thyrotoxic patients with radioiodine not all patients are successfully treated by a single dose. Multiple factors predicting radioiodine efficacy have been reported. The aim of this study was to assess whether ethnicity was associated with radioiodine response. A retrospective review was performed of patients who received radioiodine therapy for thyrotoxicosis from 1 January 2008 to 31 December 2010 and had follow-up available of a minimum of 12 months. 224 patients were included, 82.4% female, and 63.7% had Graves's disease. Radioiodine failed in 21.5% of patients overall, with a higher failure rate in the indigenous population (35.2%). When controlling for other influencing factors by logistic regression, there continued to be an increased risk for the indigenous group (OR 2.82) and those treated with antithyroid drugs following radioiodine (OR 2.04). Younger age was also associated with an increased risk of failing radioiodine therapy (OR 0.97 for each year of age). Cure rates following radioiodine were lower for indigenes independent of factors known to affect radioiodine outcome. This is the first report demonstrating ethnicity as a possible independent variable for radioiodine efficacy. Further work is needed to investigate the cause of this difference.

## 1. Introduction

Thyrotoxicosis is an important cause of morbidity and when treated inadequately may result in premature mortality [1]. Graves's disease (GD) and toxic multinodular goitre (TMNG) are the most common causes of thyrotoxicosis [2]. Despite hyperthyroidism being common, the therapeutic options have remained essentially unchanged for 50 years. It is recognised that further research in this area is needed to improve clinical outcomes [3]. For patients who require definitive treatment for GD or TMNG the treatment options remain either surgery or radioiodine (RAI). RAI has been used as treatment of thyrotoxicosis for over seven decades [4, 5]. In New Zealand, the most common definitive treatment for GD is RAI [6]. RAI enters the thyrocyte via the sodium-iodine symporter with *β* radiation resulting in cell death and reduced thyroid function. The aim of RAI is to achieve a euthyroid or hypothyroid state (with the latter corrected by thyroid hormone replacement); however not all patients become euthyroid or hypothyroid following a single dose of RAI. Successful treatment of hyperthyroidism ranges from 66 to 93% in all-cause hyperthyroidism [7–12] and from 66 to 90% in Graves's disease [13–24]. Factors reported to decrease the success of a single dose of RAI include male gender, young age, use of antithyroid medication (ATD) either before or following RAI, a large gland, severity of thyrotoxicosis at presentation, severity of thyrotoxicosis at time of treatment, uptake on scintigraphy scans prior to therapy, dose of RAI given, and presence of ophthalmopathy [7–12, 14, 16–23].

Māori are the indigenous people of New Zealand and comprised 14.6% of the population at the time of the study [25]. Our clinical impression is that Māori are more likely to have an unsuccessful response to RAI treatment than most other patients (Conaglen JV, personal communication). To date, this observation has not been formally studied. In addition, there have been no published studies assessing whether there are differences in response of thyrotoxicosis to RAI in other specific ethnic groups.

The objective of this retrospective study was to assess whether the efficacy of RAI therapy administered for thyrotoxicosis differed for Māori when compared with non-Māori and to investigate potential confounding factors that may influence differences in outcome.

## 2. Materials and Methods

A retrospective notes review was undertaken of all adult patients who received RAI (^131^I) for thyrotoxicosis at a 600-bed tertiary hospital in New Zealand, between 1 January 2008 and 31 December 2010. Participants were identified from the regional endocrine unit's database. Those patients with less than one year of follow-up data available or who had received additional definitive therapy (either RAI or thyroid surgery) prior to the start of the study period were excluded. Patients who did not receive a standard 555 MBq dose were also excluded. The study was conducted in accordance with the National Health Advisory Committee's Ethical Guidelines for Observational Studies and with permission of the local Endocrine Department.

The aetiology of the thyrotoxicosis was based on clinical, radiological, and laboratory results. Patients were considered to have GD if they had one or more of the following: diffuse symmetrical increased thyroid uptake on ^99m^technetium isotope scan, thyroid receptor antibody (TRAb) positivity, thyroid associated ophthalmopathy, or the presence of a thyroid thrill or bruit on clinical assessment. TMNG was diagnosed in those with a multinodular goitre on examination, TRAb negativity, and/or ^99m^technetium scan findings consistent with multiple nodules demonstrating increased uptake. The remaining patients had a solitary toxic adenoma (STA), diagnosed by the presence of a solitary nodule with increased uptake on ^99m^technetium scan and decreased uptake throughout the rest of the thyroid gland.

Ethnicity was determined retrospectively from hospital records, documenting patients' self-identified ethnicity on first hospital presentation, and ordered using a prioritisation method.

Data regarding pretreatment with ATD, the date ATD was started, and presence of ophthalmopathy were collected from hospital records including letters from referring clinicians. Weight measurements documented in clinical records from the day RAI therapy was administered were collected, or if no weight was documented on the day of RAI, a weight recorded within one month of RAI administration was used. Historical laboratory records were reviewed to collect the highest free T_4_ level measured in the month surrounding diagnosis. In those patients with relapsing GD, the highest free T_4_ level was taken from the month the relapse was diagnosed.

Treatment was considered successful if patients became either hypothyroid requiring L-thyroxine replacement or euthyroid and remained so to the end of follow-up. Patients who remained hyperthyroid following therapy and/or required either a subsequent dose of RAI, thyroid surgery, or long-term ATD to achieve a euthyroid state were considered failed treatment outcomes. This information was gathered from clinical records and laboratory results.

All analyses were carried out using STATA 13 (StataCorp, 2013, Stata Statistical Software: Release 13, College Station, TX, StataCorp LP). A *p* value of < 0.05 was used to reject the null hypothesis. Results are presented as mean with 95% confidence intervals or median with 5th and 95th percentiles depending on the distribution of the data. Differences between groups were analysed using chi-square, Mann-Whitney *U*, or independent *t*-tests as appropriate. Logistic regression analysis was used to determine independent factors impacting outcome with effect size presented as odds ratios and 95% confidence intervals. Variables were identified for the model using stepwise analysis with a variable cutoff of *p* < 0.05 for inclusion in the model.

## 3. Results

Two hundred and ninety-three doses of RAI were administered to 256 treatment naïve patients between 1 January 2008 and 31 December 2010. A fixed dose of 555 MBq was given to all but three patients, who received 370 MBq, and these three patients were excluded. After excluding the 30 patients for whom 365-day or more follow-up was not available, data were analysed on 223 patients.

### 3.1. General

The baseline demographics are presented in Table 1. The majority of patients were female (82.5%), with a median age of 53.7 years. Thirty-two percent of patients recorded Māori as their ethnicity. Of the 63.7% classified as non-Māori, 111 were recorded as New Zealand European with the remainder comprising 14 Asians, 13 Other Europeans, 3 Pacific Islanders, and 1 African. The 10 individuals in whom ethnicity was not stated were excluded from analyses where ethnicity was a variable. GD was diagnosed in 63.7% and TMNG in 33.6%. The majority of patients (87%) received ATD prior to RAI for a median length of 49 weeks; most received carbimazole with only 6 individuals receiving propylthiouracil. All patients stopped ATD therapy at least 5 days prior to RAI. The most common reasons for not receiving ATD were mild/subclinical disease (42.9%) or adverse reactions to ATD (39.3%). Patients were followed up for a median duration of 913 days.

### 3.2. Māori Ethnicity

The demographics of Māori were different when compared to non-Māori (Table 2). Māori participants were younger (*p* = 0.0132), more likely to have a TMNG (*p* = 0.011), and more likely to receive ATDs prior to RAI therapy (*p* = 0.048) than non-Māori. There was no difference in gender distribution, body weight, free T_4_ at presentation, presence of ophthalmopathy (in GD patients), median length of follow-up, or use of ATD after RAI.

### 3.3. Outcomes

At the end of follow-up 78.5% of patients became either hypothyroid or euthyroid (Table 3). Factors influencing outcome are reported in Table 4. Patients who had persistent thyrotoxicosis following RAI were 5.9 years younger (*p* = 0.012) and more likely to be Māori (54.4% versus 27.5%, *p* = 0.001) than those who had achieved a successful outcome. Patients in whom RAI therapy failed had at diagnosis an FT_4_ level that was 12.45 pmol/L higher than those who were successfully treated (*p* ≤ 0.0005) and were more likely to be pretreated with ATDs (97.9% versus 84.0%, *p* = 0.011). In those patients who received ATD therapy prior to RAI, there was no difference in the length of treatment in those who remained thyrotoxic following RAI as compared to those who became either euthyroid or hypothyroid (44 weeks and 57 weeks, resp., *p* = 0.2533). Treatment with ATD therapy following RAI was associated with a higher failure rate (53.2% and 33.8%, *p* = 0.018). There was no effect of gender (*p* = 0.109), aetiology (GD versus TMNG) (*p* = 0.185), weight at treatment (*p* = 0.6674), presence of ophthalmology (*p* = 0.461), or follow-up length (*p* = 0.179) on outcome.

### 3.4. Independent Variables

Using stepwise regression analysis, variables with *p* value < 0.05 were identified for the model. Thus age, ethnicity, and treatment with ATD following RAI were all identified for inclusion in the model. ATD therapy prior to RAI was unable to be included in the model as it correctly predicted persistent thyrotoxicosis in all but two individuals. Diagnosis, weight, gender, FT_4_ level at presentation, and length of ATD therapy following RAI were not significant and were not included in the model.

The logistic regression analysis is presented in Table 5. This analysis showed that even when controlling for other influencing factors, there continued to be an increased risk for Māori (OR 2.82) and for those treated with ATD following RAI (OR 2.04). There was also an association of increased risk of failing RAI therapy with younger age (OR 0.97 for each year of age).

## 4. Discussion

Radioiodine has been used for the treatment of hyperthyroidism for over 70 years with the aim of curing thyrotoxicosis [4, 5]. The efficacy of RAI in curing thyrotoxicosis varies from 50 to 90% [26]; in this study 78.5% of patients were cured by a single dose. Māori patients experienced an increased treatment failure following a single dose of RAI and this disparity in outcome continued after controlling for other variables reported to affect outcome. Young age and severity at presentation have been reported previously as factors that can negatively influence outcomes following RAI and were also identified in this study [7–9, 11, 12, 22–24]. However, after stepwise regression analysis was performed, severity at presentation was no longer found to be associated with outcome. Pretreatment with ATD, length of ATD use, and ATD following RAI were also associated with therapy failure. Prior use of ATD has been shown to increase failure rates in some observational studies [7, 8, 17, 22]; however this was not found in randomised controlled trials investigating the effect of ATD prior to RAI [27, 28]. Previous findings of male gender and ophthalmopathy influencing failure of treatment [9, 12, 24] were not associated with altered outcome in the current study.

In order to better understand the factors that influence this outcome difference for Māori other known variables available were controlled for. In univariate analysis, Māori showed a higher risk of treatment failure compared to non-Māori (OR 3.13, *p* = 0.001) and when other available confounding factors were controlled for, the increased risk reduced but persisted for Māori (OR 2.61, *p* = 0.010). It is unclear why RAI was less successful in the treatment of Māori. In this study, some of the effect can be explained by young age and use of ATD following RAI. However, the elevated odds ratio following control of these factors indicates other, as yet, unexplained factors at play.

Ethnic variations in treatment response to other forms of therapy for hyperthyroidism have been reported. African American patients with GD were shown to be less likely to have resolution of thyrotoxicosis with ATD when compared to non-Hispanic Whites (72.7% versus 97.0%) [29]. However, this is the first report to demonstrate an ethnic disparity in the outcome of treating thyrotoxicosis with RAI. One limitation is that this is a retrospective study, so other factors potentially influencing RAI outcome such as percent ^131^I uptake and thyroid volume were not available. Data on other variables, such as socioeconomic factors, barriers to care, and dietary iodine exposure, were also unavailable and these may play a role in differences in outcome. Traditionally, Māori had a diet high in seafood which may have resulted in a higher iodine intake when compared to non-Māori; however increased urbanization and decreased socioeconomic status have altered access to such traditional diets. Currently we do not have data on differences in dietary iodine exposure between these two groups. Whether the observed differences in outcome to RAI are clinical, social (including dietary), or a combination of both, these results emphasise the need for further research to identify why RAI outcome is poorer for Māori than non-Māori.

Standard practice in managing patients with thyrotoxicosis who fail initial RAI therapy is to give additional doses. This leads to a higher cumulative dose of RAI for these patients. The higher doses of RAI used in management of thyroid cancer are recognised to be associated with an increased risk of a second malignancy [30]. Data on the risk at the lower doses used for treating thyrotoxicosis have been conflicting. A Finnish study of 2793 patients suggested that the lower doses of RAI used for thyrotoxicosis may be associated with an increased risk of treatment-associated malignancy, particularly breast, stomach, and kidney cancer, and that the risk of malignancy is associated with an increased cumulative dose of RAI [31]. However, a more recent study by the same group suggested that cancer risk in thyrotoxicosis is not affected by treatment modality; rather any increased cancer risk may be related to thyrotoxicosis per se or other risk factors [32]. If the earlier Finnish findings were correct, the higher cumulative RAI doses for Māori may be of clinical significance. A potential increase in breast and stomach cancer is of particular concern given that these two malignancies are among the five most commonly occurring cancers for Māori as well as being two of the most common causes of cancer death for Māori [33].

## 5. Conclusions

RAI is the most common definitive treatment for thyrotoxicosis in New Zealand. While RAI is successful therapy for the majority of patients, approximately one in five patients still fail to respond to one treatment. Many studies have investigated factors that influence the efficacy of RAI; however this study demonstrates that for Māori, a specific indigenous ethnic group, treatment with RAI is not as effective as when administered to the rest of the population. More work is needed to better understand the factors influencing this inequity to improve health outcomes for all.

## Figures and Tables

**Table 1:** Baseline demographic data.

	*n*		Total
Age	223		53.7 (51.8, 55.6)

Gender	223	F	184 (82.5%)
		M	39 (17.5%)

Ethnicity	213	Māori	71 (31.9%)
		NMāori	142 (63.6%)

Diagnosis	223	Graves's	142 (63.6%)
		TMNG	75 (33.6%)
		STA	6 (3.7%)

Median weight (kg)	208		74.6 (52.2, 118.8)

Ophthalmopathy	140	Nil	103 (73.57%)
		Mild	26 (18.57%)
		Mod.	7 (5.00%)
		Sev.	4 (2.86%)

T_4_ at presentation	210		29 (12, 65.4)

Pretreatment ATD	223		184 (86.4%)

Length of ATD (wks)	193		49 (13, 439)

Posttreatment ATD	192		74 (38.54%)

Length of FU (wks)	223		913 (432, 1466)

ATD: antithyroid medication; F: female; M: male; NMāori: non-Māori; GD: Graves's disease; TMNG: toxic multinodular goitre; STA: solitary toxic adenoma; Mod.: moderate eye disease; Sev.: severe eye disease.

Continuous variables are expressed as mean (95% confidence intervals) or median (5th, 95th percentile) according to their distribution. Categorical variables are expressed as number (percentage).

**Table 2:** Baseline data by ethnicity.

		Non-Māori (*n* = 142)		Māori (*n* = 71)		*p*
		*n*		*n*		
Age		142	55 (52.6, 57.6)	71	50.4 (47.7, 53.1)	0.0132

Gender	F	142	116 (81.7%)	71	61 (85.9%)	0.438
	M		26 (18.3%)		10 (14.1%)	

Diagnosis	GD	142	98 (69.0%)	71	36 (50.7%)	0.011
	TMNG		39 (27.5%)		34 (47.9%)	
	STA		5 (3.5%)		1 (1.4%)	

Weight (kg)		133	73.7 (52.2, 118.3)	67	81.4 (55.1, 118.1)	0.0698

Ophthalmopathy	Nil	96	71 (74.0%)	36	25 (69.4%)	0.814
	Mild		18 (18.8%)		7 (19.4%)	
	Mod.		4 (4.2%)		3 (8.3%)	
	Sev.		3 (3.0%)		1 (2.8%)	

T_4_ at presentation		133	28 (12, 61)	68	30 (11, 74)	0.2461

Pretreatment ATD		142	118 (83.1%)	71	66 (93.0%)	0.048

Length of ATD (wks)		117	44 (9, 374)	66	52 (14, 504)	0.4837

Posttreatment ATD		117	42 (35.9%)	65	28 (43.08%)	0.34

Follow-up length (wks)		142	944 (428, 1459)	71	791 (432, 1481)	0.1361

ATD: antithyroid medication; F: female; M: male; GD: Graves's disease; TMNG: toxic multinodular goitre; STA: solitary toxic adenoma; Mod.: moderate eye disease; Sev.: severe eye disease.

Continuous variables are expressed as mean (95% confidence intervals) or median (5th, 95th percentile) according to their distribution. Categorical variables are expressed as number (percentage).

**Table 3:** Outcomes following radioactive iodine.

		Non-Māori		Māori		*p*
		*n*	%	*n*	%	
Overall	Failure	21	14.8%	25	35.2%	0.001
	Success	121	85.2%	46	64.8%	

GD	Success	82	83.7%	20	55.6%	0.001
	Failure	16	16.3%	16	44.4%	

TMNG/STA	Success	39	88.6%	26	74.3%	0.097
	Failure	5	11.4%	9	25.7%	

GD: Graves's disease; TMNG: toxic multinodular goitre; STA: solitary toxic adenoma.

**Table 4:** Factors influencing radioactive iodine outcome.

		Success		Failure		*p*
		*n*		*n*		
Age		175	55.0 (52.8, 57.2)	48	49.1 (45.3, 52.9)	0.0117

Gender	F	175	148 (84.6%)	48	36 (75.0%)	0.122
	M		27 (15.4%)		12 (25.0%)	

Diagnosis	GD	175	109 (62.3%)	48	33 (68.8%)	0.369
	TMNG		60 (34.3%)		15 (31.2%)	
	STA		6 (3.4%)		0 (0%)	

Ethnicity	NMāori	167	121 (72.5%)	46	21 (45.6%)	0.001
	Māori		46 (27.5%)		25 (54.4%)	

Weight (kg)		161	74.5 (52.2, 118)	47	75.2 (53.8, 118.1)	0.6674

Ophthalmopathy	Nil	106	81 (76.4%)	34	22 (64.7%)	
	Mild		18 (17.0%)		8 (23.5%)	
	Mod.		5 (4.7%)		2 (5.9%)	
	Sev.		2 (1.9%)		2 (5.9%)	0.461

T_4_ at presentation		164	27.3 (12.0, 60.1)	46	39.8 (16.9, 68.0)	0.0003

Pretreatment ATD		175	147 (84.0%)	48	47 (97.9%)	0.011

Length of pretreatment (wks)		146	44 (13, 335)	47	57 (13, 556)	0.2533

Posttreatment ATD		145	49 (33.79%)	47	25 (53.19%)	0.018

Length of follow-up (wks)		175	875 (419, 1466)	48	961 (456, 1483)	0.1148

ATD: antithyroid medication; F: female; M: male; GD: Graves's disease; TMNG: toxic multinodular goitre; STA: solitary toxic adenoma; NMāori: non-Māori; Mod.: moderate eye disease; Sev.: severe eye disease.

Continuous variables are expressed as mean (95% confidence intervals) or median (5th, 95th percentile) according to their distribution. Categorical variables are expressed as number (percentage).

**Table 5:** Logistic regression analysis of factors influencing radioactive iodine outcome.

	OR	95% CI	*p*
Age (per yr)	0.96	0.93, 0.99	0.015
Māori ethnicity	2.60	1.26, 5.35	0.010
Posttreatment ATD	2.19	1.06, 4.54	0.033

ATD: antithyroid medication; OR: odds ratio; CI: confidence interval.

Logistic regression model of variables shown to have *p* < 0.05 in stepwise analysis. Variable of pretreatment was omitted in analysis due to estimability (near-perfect prediction of failing therapy).
